# Association Study with 77 SNPs Confirms the Robust Role for the rs10830963/*G* of *MTNR1B* Variant and Identifies Two Novel Associations in Gestational Diabetes Mellitus Development

**DOI:** 10.1371/journal.pone.0169781

**Published:** 2017-01-10

**Authors:** Klara Rosta, Zahra Al-Aissa, Orsolya Hadarits, Jürgen Harreiter, Ákos Nádasdi, Fanni Kelemen, Dagmar Bancher-Todesca, Zsolt Komlósi, László Németh, János Rigó, István Sziller, Anikó Somogyi, Alexandra Kautzky-Willer, Gábor Firneisz

**Affiliations:** 1 Department of Obstetrics and Gynecology, Medical University of Vienna, Vienna, Austria; 2 1^st^ Department of Obstetrics and Gynecology, Semmelweis University, Budapest, Hungary; 3 2^nd^ Department of Internal Medicine, Semmelweis University, Budapest, Hungary; 4 Gender Medicine Unit, Division of Endocrinology and Metabolism, Department of Internal Medicine III, Medical University of Vienna, Vienna, Austria; 5 University of Szeged, Faculty of Medicine, Szeged, Hungary; 6 Department of Pulmonology, Semmelweis University, Budapest, Hungary; 7 Department of Probability Theory and Statistics, Eötvös Loránd University, Budapest, Hungary; 8 Department of Obstetrics and Gynecology, Szent Imre Teaching Hospital, Budapest, Hungary; 9 Hungarian Academy of Sciences - Semmelweis University, Molecular Medicine Research Group, Budapest, Hungary; University of Oslo, NORWAY

## Abstract

**Context:**

Genetic variation in human maternal DNA contributes to the susceptibility for development of gestational diabetes mellitus (GDM).

**Objective:**

We assessed 77 maternal single nucleotide gene polymorphisms (SNPs) for associations with GDM or plasma glucose levels at OGTT in pregnancy.

**Methods:**

960 pregnant women (after dropouts 820: case/control: m99’WHO: 303/517, IADPSG: 287/533) were enrolled in two countries into this case-control study. After genomic DNA isolation the 820 samples were collected in a GDM biobank and assessed using KASP (LGC Genomics) genotyping assay. Logistic regression risk models were used to calculate ORs according to IADPSG/m’99WHO criteria based on standard OGTT values.

**Results:**

The most important risk alleles associated with GDM were rs10830963/*G* of *MTNR1B* (OR = 1.84/1.64 [IADPSG/m’99WHO], p = 0.0007/0.006), rs7754840/*C* (OR = 1.51/NS, p = 0.016) of *CDKAL1* and rs1799884/*T* (OR = 1.4/1.56, p = 0.04/0.006) of GCK. The rs13266634/*T* (*SLC30A8*, OR = 0.74/0.71, p = 0.05/0.02) and rs7578326/*G* (*LOC646736/IRS1*, OR = 0.62/0.60, p = 0.001/0.006) variants were associated with lower risk to develop GDM. Carrying a minor allele of rs10830963 (*MTNR1B*); rs7903146 (*TCF7L2*); rs1799884 (*GCK*) SNPs were associated with increased plasma glucose levels at routine OGTT.

**Conclusions:**

We confirmed the robust association of *MTNR1B* rs10830963/*G* variant with GDM binary and glycemic traits in this Caucasian case-control study. As novel associations we report the minor, *G* allele of the rs7578326 SNP in the *LOC646736/IRS1* region as a significant and the rs13266634/*T* SNP (*SLC30A8)* as a suggestive protective variant against GDM development. Genetic susceptibility appears to be more preponderant in individuals who meet both the modified 99’WHO and the IADPSG GDM diagnostic criteria.

## Introduction

Abnormal glucose tolerance first recognized during pregnancy is defined as gestational diabetes mellitus (GDM). The estimated prevalence of GDM varies according to the study population and diagnostic criteria. A plethora of different GDM classification methods and diagnostic guidelines are available [1]. Prevalence estimates of gestational diabetes mellitus indicate that it is a common disease in developed countries with a prevalence ranging from 8.1–14.8% in Hungary (HUN) [2] and >10% in Austria (AT) [1], depending on the diagnostic criteria applied.

Pregnancy is generally characterized by increased adiposity and increased insulin resistance. Insulin resistance during pregnancy occurs partially due to the increased production of human placental lactogen, estrogen and prolactin especially in the second and third trimesters and also due to weight gain. Individuals with limited beta cell response to compensate for physiologically increased insulin resistance are more prone to develop GDM.

Due to the trends in modern societies the more prevalent clinical risk factors, such as higher pre-pregnancy body mass index (BMI) [3] and advanced age [4] at pregnancy resulted in increased insulin- resistance and a need for a compensatory increase in beta cell response. In line with this the prevalence of GDM started to increase sharply in the early 90s [4].

The role for genetic factors in disease development is confirmed by findings that women with a diabetic sibling have an 8.4-fold increased risk of GDM [5]. Since beta cell dysfunction is known to play a critical role in GDM [6, 7] recent evidence from a twin study demonstrating a 75% heritability in insulin secretion variability in younger adults [8] underlines the importance of genetic components in the development of GDM.

As GDM and type 2 diabetes mellitus (T2D) share similarities in their pathogenesis with respect to impaired insulin secretion and increased insulin resistance, research efforts focused on mapping their genetic properties. These studies demonstrated that GDM and T2D share common genetic background, with similar magnitude of effect sizes on the same risk alleles [9]. A few authors even suggested that GDM and type 2 diabetes are two aspects of the same entity [10]

Despite the number of genome wide associations studies (GWAS) performed and reports on associations for selected genotypes in GDM, the protective and risk genetic loci of GDM are still not fully elucidated [9, 11–17]. This might be due to differences in the study populations (ethnicity), small sample size and the different diagnostic criteria used. Most of the GWAS studies in this disease were conducted in Asian populations [11], highlighting the need for confirmation in Caucasian populations [18].

In our study, we aimed to identify maternal gene variants that are associated with GDM or influence fasting and 2-hour glucose levels after a routine 75 g oral glucose tolerance test (OGTT) during pregnancy (24-28^th^ gestational week [gw]). A set of 77 gene variants single nucleotide polymorphisms (SNPs) were selected and the majority of these variants were common variants previously reported to be associated with either T2D, GDM or with important metabolic traits, such as hemoglobin A1c levels, fasting and 2 hour plasma glucose levels, insulin-resistance indices, proinsulin levels or with other related traits such as the BMI [19]. We compared the impact of two different diagnostic criteria of GDM on the association of gene variants with GDM. In Austria, we used the International Association of the Diabetes and Pregnancy Study Groups (IADPSG) criteria [GDM: 75gCH OGTT at 24–28 gw: fasting plasma glucose (FPG)≥5.1mmol/L, 60 min plasma glucose (PG)≥10.0 mmol/L, 120 min PG≥8.5 mmol/L], while the Hungarian centers used the modified 1999 World Health Organization (WHO) recommendation [GDM: 75gCH OGTT at 24–28 gw: FPG≥6.1 mmol/L, 120 min PG≥7.8 mmol/L].

We assessed T2D susceptibility gene variants to confirm the interrelated genetic background of GDM in a Caucasian population. We also hypothesized, that patient selection by different diagnostic criteria in GDM might influence the results of the genetic association study, as different threshold values in OGTT can have an influence on patient distribution between the control (CNTRL) and GDM group and thus could represent a bias if remains without adjustment.

Our results support the conception of a similar genetic background to GDM and T2D, however there are variances in the effect sizes of the risk/protective SNPs in between the two diseases. We confirmed the robust role of rs10830963/*G* of Melatonin receptor 1B *(MTNR1B)* gene variant in developing GDM or glycemic traits. Furthermore, we identified two novel associations, namely the minor, *G* allele of the rs7578326 SNP in the *LOC646736/IRS1* region as a significant, and the rs13266634/*T* SNP Solute carrier family 30 member 8 (*SLC30A8*) as a suggestive protective variant against GDM development. According to our findings, the minor allele frequency of the known common causal *MTNR1B* variant, rs10830963 is significantly higher in the GDM subgroup which meet both the modified 99’ WHO and the IADPSG diagnostic criteria.

## Materials and Methods

### Patients

The institutional (Semmelweis University, Regional and Institutional Committee of Science and Research Ethics and Ethics Committee of the Medical University of Vienna) and the national ethical bodies of Hungary (Medical Research Council Scientific and Research Committee) and Austria (Federal Office for Safety and Health care) have approved the study, which was conducted according to the declaration of Helsinki. Pregnant women were enrolled to the study after signing the informed consent for the whole project and a special consent for storing their samples in a biobank for use in anonymized genetic studies.

Patients were recruited from two Hungarian and a single Austrian centers in the framework of the European Foundation for the Study of Diabetes New Horizons initiative.

### Inclusion criteria

The diagnosis of GDM (inclusion criteria) has been established according to the national recommendations of the two countries: the Austrian study center applied the IADPSG criteria [GDM: 75gCH OGTT at 24–28 gw: FPG≥5.1mmol/L, 60 min PG≥10.0 mmol/L, 120 min PG≥8.5 mmol/L], while the Hungarian centers used the modified 1999 WHO recommendation [GDM: 75gCH OGTT at 24–28 gw: FPG≥6.1 mmol/L, 120 min PG≥7.8 mmol/L] [20, 21].

### Exclusion criteria

Exclusion criteria were based in part on the STrengthening the Reporting of observational (case-control) studies in Epidemiology statement for Genetic Association studies (STREGA) guidelines [22]. In addition we excluded patients with: diabetes in pregnancy (i.e. overt diabetes)and diabetes categories other than GDM (e.g.: autoimmune), any apparent major disease or chronically treated with medication with known impact on glycemic control, in vitro fertilization, missing major clinical data, twin pregnancies or variables likely to be associated with population stratification (e.g.: non-European ancestry).

### Clinical data collection

We collected all relevant clinical information that was used in the case- control study or qualitative trait assessments, including the result of the OGTT, pre-pregnancy BMI, maternal age and the birth-weight percentiles [23].

### Research design

As two different diagnostic GDM criteria were applied (IADPSG or the modified 99' WHO) in the two participating countries the following protocol was employed for the analysis of genotype associations: we first had to reclassify the entire dataset of the whole study population according to both criteria based on the OGTT results (FPG and 2 hour PG values) as a ‘diagnosis stratification procedure’. This was possible since there was no medical intervention prior to the 75g OGTT and the test itself was performed in the standardized time-frame of gestation (24^th^-28^th^ gestational week). Those individuals in whom the GDM diagnosis was established exclusively on the basis of the 60 min OGTT result remained in the GDM group in the ‘diagnosis stratification procedure’. Such cases are rare (less than 5.7% according to the Hyperglycemia and Adverse Pregnancy Outcomes (HAPO) Study [24] and 13% in the participating Austrian study population). We have performed all binary analysis twice, first using the 99’ modified WHO criteria and second using the IADPSG criteria.

A set of 77 SNPs were selected based on the results of prior genome wide association studies (GWAS) on T2D, BMI, Insulin resistance (IR), Insulin secretion/ beta cell function, plasma glucose or serum insulin level traits. Functionally, the reported genes were suggested to be implicated in the incretin effect, beta-cell function or genesis, potassium channel function, amyloid formation, zinc transport, insulin resistance, obesity development, insulin-like growth factor (IGF) system, vessel formation, glucose homeostasis, circadian rhythm, neuronal regulation of appetite, energy balance or immunoregulation (S1 Table).

Subsequently we provided two numerically different odds ratios and p-values for each gene variants that were significantly associated with GDM according to the corresponding diagnostic system applied in the analysis.

### Genomic DNA isolation and genotyping

Genomic DNA was isolated using a magnetic bead based robotized approach (Hamilton Robotics, Magna Starlet, Bonaduz, Switzerland) from EDTA-anticoagulated whole blood samples obtained from cubital veins. Kompetitive Allele Specific PCR (KASP^™^) genotyping (FRET [fluorescence resonant energy transfer] based assay) (LGC Genomics, Teddington, Middlesex, UK) was used for the bi-allelic discrimination of 77 SNPs. The overall call rate for all SNPs assessed exceeded the 97% and no discordant genotypes were identified in the control samples of which genomic DNA were isolated in two different runs and subsequently genotyped separately in duplicates for all SNPs. Results were presented using the SNP viewer software (version 1.99, Hoddesdon, UK) and genotype data were extracted for statistical analysis.

### Statistical analysis

#### GDM as binary trait

All statistical analyses of this case-control study were carried out in a specially designed program in R-project language. We first analyzed associations between genotypes according to the 77 assessed SNPs and the diagnosis of gestational diabetes mellitus as a case-control study. As the diagnosis of GDM was originally established according to criteria (IADPSG or modified 99' WHO) which was used in the center of the patient care provider, we first had to reclassify the entire dataset of the whole study population according to both criteria based on the OGTT results (FPG and 2 hour PG values) as a ‘diagnosis stratification procedure’. Subsequently, we analyzed the data using the logistic regression method under both the dominant and the additive genetic models. We calculated odds ratio (OR), statistical power and p-value for every SNP and used the Benjamini-Hochberg p-correction method to minimize false discovery. ORs were reflective for the Effect Sizes in our case-control study for binary outcomes and were calculated to represent the effects of carrying the reported minor alleles under the dominant model or the effect of the minor allele under the additive genetic model (S2 Table). Subsequently minor allele frequencies were also calculated for each 77 SNPs assessed (S3 Table).

We adjusted our result to the maternal age and pre-pregnancy BMI (S2 Table, Panel A) as covariates in both models, provided that there were no published prior data or direct result in this analysis for the association between the assessed SNPs and pre-pregnancy BMI. If an association between the SNP assessed and pre-pregnancy BMI was observed then only age was used as covariate in adjustment calculations.

Due to that an effect through the causative chain still can be a potential risk for disease development and that BMI-adjustment might possibly be important even if there is a significant SNP-BMI association we reported the p-values and ORs for each 77 SNPs association with GDM one adjusted for age (S2 Table, Panel B) and additionally adjusted for age and pre-pregnancy BMI also (S2 Table, Panel A). However the literature suggests to use the adjusted data by age and BMI as these are often neglected confounders and concludes that this approach results in a more accurate analysis compared to the direct use of unadjusted raw data [18]. Therefore—after exclusion of the causative chain for our most significant findings—we indicate these genetic association results adjusted by age and BMI. Deviations from the Hardy-Weinberg equilibrium in the genotype distributions were assessed for all SNPs using Chi Squared test (S4 Table). We used post-hoc analysis for power calculation. From the p value and statistical power we calculated the exact probability of the existing effect for each gene variant associated significantly with GDM.

#### Glycemic traits

In addition we performed analysis for the associations between genotypes according to 77 assessed SNPs and glycemic traits, such as the fasting and 2 hour plasma glucose levels at OGTT and pre-pregnancy BMI using linear regression both under the additive and also under the dominant genetic models. Coefficient values were calculated for significantly associated SNPs in the glycemic trait analysis representing effect sizes both on the fasting and the 2-hour plasma glucose levels and expressed in mmol/L.

To further improve the applied statistics we also re-analyzed our data using a bootstrap resampling method for the linear regression to confirm results when the glycemic traits were analyzed. The bootstrap application was used due to that it is a statistically appropriate way to control and check the stability of the results.

All data were assessed according to the guidelines of STREGA which is an extension of the STrengthening the Reporting of OBservational studies in Epidemiology (STROBE) statement [22].

#### Clinical characteristics

Both the Shapiro—Wilks and the Kolmogorov—Smirnov tests were used to assess normality. Mann—Whitney U-test (MWU) was used to compare means and detect differences in case of nonparametric distributions, and 2-tailed t-test with independent variables was used when the distribution was normal. For this re-classified analysis of clinical data we have used Welch-test for comparisons between the countries as the standard deviation (SD) values were not known (and distributions of these parameters are usually asymptotic normal in literature). The, U‘ test was performed with two-sided counterhypothesis due to that we did not hypothesize difference between the two countries.

## Results

Altogether 960 pregnant women were enrolled to the study. Dropouts due to insufficient sampling and withdrawn consent eventually reduced the final sample size to 820. Out of the 820, 303 would be diagnosed with GDM according to the 99’ modified WHO criteria and 287 according to the IADPSG criteria. The clinical characteristics of the pregnant population studied are indicated in Table 1.

**Table 1:** Clinical characteristics of the pregnant population studied.

		75g CH OGTT plasma glucose values in GDM group (mM)			75g CH OGTT plasma glucose values in Control group (mM)			Pre-pregnancy BMI (kg/m^2^)		Age at delivery (years)		Weight gain during pregnancy (kg)		HbA1c %^≠^ (IFCC Unit-mmol/mol)
		0’	60’^#^	120’	0’	60’^#^	120’	GDM	Control	GDM	Control	GDM	Control	GDM
**Austria**	Mean	5.14 *°	9.68 *	7.38 *°	4.38 *°	6.80 *	5.42 *	28.31 *	23.40 *	32.04^+^°	30.51 *	9.68	9.47°	5.30 (34)
n = 183/147 (Cntrl/GDM)	95%CI of the Difference (between GDM and Cntrl study groups)	0.63–0.87	2.47–3.28	1.61–2.30	0.63–0.87	2.47–3.28	1.61–2.30	2.72–7.09		0.08–2.97		-1.63–2.04		95%CI
														(GDM only):
														5.21–5.38
														(33–35)
**Hungary**	Mean	4.96 *°	NA	8.72*°	4.52*°	NA	5.45*	26.78*	23.32*	33.70*°	31.25*	8.72*	13.80*°	5.20 (33)
n = 408/195 (Cntrl/GDM)	95%CI of the Difference (between GDM and Cntrl study groups)	0.34–0.54	NA	3.06–3.47	0.34–0.54	NA	0.34–0.54	2.55–4.36		1.54–3.36		-6.07–-4.07		95%CI
														(GDM only):
														5.10–5.30
														(32–34)

Significant differences were found between the GDM and the Control groups as follows:

*p<10^−4^;

^+^p<0.05 (t—test or MWU).

Significant differences found between the Hungarian and Austrian study populations

° p<0.05.

^#^ 60’ plasma glucose values at OGTT were only assessed in Austria.

^≠^ HbA1c values were only determined in patients with GDM, but not in the controls.

The clinical data (0 min and 120 min plasma glucose values at 75g OGTT, pre-pregnancy BMI, age) were also reclassified according both the IADPSG and m99‘ WHO criteria for the entire (joint Austrian and Hungarian) study population (S5 Table).

In addition to the differences in the plasma glucose values at OGTT significant differences were detected in pre-pregnancy BMI and age at delivery between the GDM and control populations in both countries. We also found differences in the 0’ PG values (HUN>AT, 4.52mM vs. 4.38mM, p<0.05), weight gain during pregnancy in the controls (HUN>AT 13.80kg vs. 9.47kg, p<0.05) and in the 0’ PG (AT>HUN 5.14mM vs. 4.96mM, p<0.05) and 120’ PG (HUN>AT 8.72mM vs.7.38mM, p<0.05) values and age at delivery (HUN>AT 33.7 years vs. 32.04 years, p< 0.05) between the GDM groups of the two countries. However differences in the clinical characteristics between Austria and Hungary are unlikely to affect the genetic study as the clinical characteristics were compared using the country designated diagnosis as grouping variable, in contrast to the genetic analysis, when we regrouped the study population based on the 75g OGTT results according to both GDM diagnostic criteria.

Associations found at significant or suggestive levels in the case-control analysis are indicated in Table 2. There was no significant deviation from the Hardy-Weinberg equilibrium (S4 Table) in the genotype distributions of SNPs with significant results.

**Table 2:** Association between maternal gene variants and gestational diabetes mellitus (GDM) using both the International Association of Diabetes and Pregnancy Study Group (IADPSG) and the modified 99' World Health Organization (WHO) GDM diagnostic criteria.

SNP—(major / minor allele)	Reported Gene (HGNC Symbol)	SNP locus (chr: base)°	Functional class	Gene function	Prior GWAS	Prior assn. studies	MAF in GDM cases IADPSG / m99' WHO	MAF in Cntrl IADPSG/ m99' WHO	MAF in 1000 Genomes in EU	p IADPSG / m99’ WHO	OR IADPSG / m99' WHO	Effect size IADPSG/ m99’ WHO	Model	Probabi-lity of existing effect IADPSG / m99' WHO	Statistical Power IADPSG / m99' WHO
rs10830963 *(C/G)*	*MTNR1B*	11: 92975544	intron variant	Encodes one of two high affinity forms of a receptor for melatonin. Melatonin. may regulate glucose metabolism by affecting circadian and first phase insulin secretion.	GDM	GDM	0.36 / 0.36	0.28 / 0.28	0.29	0.0007^a^ / 0.006	**1.84/1.64** (95% CI: 1.54–2.21 / 1.38–1.97)	0.308 / 0.249	D	>99% / 99%	0.9/0.73
					[11, 14] FPG levels	[15]									
					[25]	T2D									
					T2D	[9]									
					[26]										
rs7578326 (A/G)*	LOC 646736 / IRS1*	2: 226155937	lnc RNA (RNA gene) variant	Unknown	T2D	IFG T2D MetS	0.31 / 0.32	0.39 / 0.38	0.35	0.001^a^/0.006	**0.62/0.60** (95% CI: 0.46–0.67 / 0.51–0.73)	0.285 / 0.247	D	>99% / 99%	0.65/0.65
					[27]	[28]									
rs1799884 *(C/T)*	*GCK*	7:44189469	intron variant	GCK (Glucokinase (Hexokinase 4)) encodes an enzyme that catalyzes the initial step in utilization of glucose by the beta-cell and liver at physiological glucose concentration.	HbA1c	GDM	0.17 / 0.18	0.15 / 0.14	0.18	0.04/0.006	**1.4/1.56** (95% CI: 1.18–1.65 / 1.34–1.84)	0.173 / 0.229	A	93% / 99%	(0.51/0.7)
					[29]	[13]									
rs7754840 *(G/C)*	*CDKAL1*	6: 20661019	intron variant	A member of the methyl-thiotransferase family. Protein translation. insulin synthesis. Early phase insulin response.	GDM	Meta-analysis T2D	0.33 / 0.32	0.30 / 0.31	0.32	0.016^b^/NS	**1.51**^b^/NS (95% CI: 1.26–1.79 / NA)	0.147 / NR	D	97% / NA	0.61/NA
					[11]	[32]									
					T2D										
					[26, 30–32]										
rs13266634 (C/T)**	SLC30A8**	8: 117172544	intron variant. missense	Encodes a zinc transporter involved in the accumulation of zinc in intracellular vesicles. High level only in the pancreas, particularly in islets of Langerhans in Insulin-secreting cells.	2TD	Reduced first-phase insulin release in T2D offspring	0.25/0.24	0.30/0.30	0.28	0.05/ 0.02	**0.74/0.71** (95% CI: 0.65–0.87 / 0.61–0.82)	0.188 / 0.220	A	88% /95%	0.36/0.42
					[32] HbA1c levels	[34]									
					[27, 33]										

Effect of minor alleles of the corresponding single nucleotide polymorphism on disease development as odds ratios (OR).

p values indicated are after adjustment to pre-pregnancy body mass index (BMI) and age covariates.

Prior genome wide association studies and reported gene functions and are summarized

D = dominant genetic model

A = additive genetic model

^a^ Significant after adjustment to age and BMI and Benjamini-Hochberg correction.

^b^ Adjustment to age as covariate only (due to significant association with the pre-pregnancy BMI).

* Novel significant genetic association reported in GDM disease.

** Novel suggestive genetic association reported in GDM disease.

° To indicate the chromosomal position the NCBI SNP database (release: 107.) was used.

### GDM as binary trait

#### Association of *MTNR1B* rs10830963 with GDM

The most significant findings were related to the rs10830963 polymorphism of the melatonin receptor 1B (MTNR1B): carrying a *G* allele of the rs10830963 polymorphism of the *MTNR1B* significantly increased the risk of developing GDM (Table 2) in our case-control study.

The associations of rs10830963 with GDM binary trait resulted in better significance (IADPSG / m99'WHO p: 7x10^-4^/6x10^-3^) and higher odds ratios (ORs: 1.84/1.64) under the dominant model compared to the additive model (p: 0.003/0.012, OR: 1.48/1.39, respectively) after adjustment to maternal age and pre-pregnancy BMI covariates. This association remained significant under the dominant model, when adjusted p-values were further corrected using the Benjamini-Hochberg (B-H) method. Accordingly, minor allele frequencies (MAF) were higher in the GDM groups than in the control groups under each diagnostic criteria applied (IADPSG: 36% vs. 28%, m99’WHO: 36% vs. 28%; GDM vs. Control groups respectively, Table 2).

**Fig 1:**
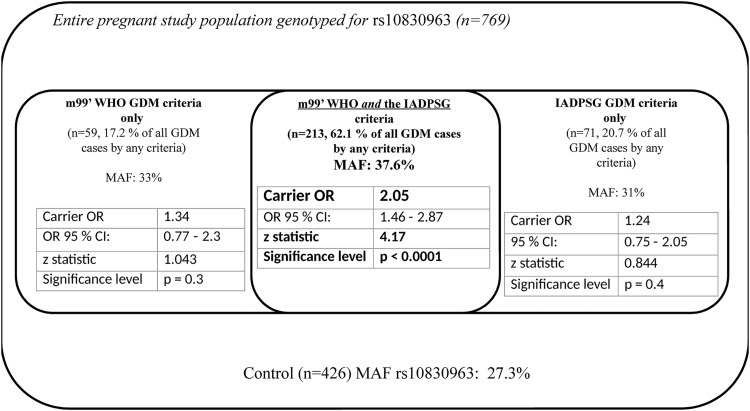
*MTNR1B* rs10830963 (true causal gene variant, risk allele *G*) associated odds ratios of developing GDM by different diagnostic criteria.

Carrying the *G* risk allele of the *MTNR1B* rs10830963 in any form was associated with substantially higher odds of disease development in the group of patients who could meet both the m99’ WHO and the IADPSG criteria (OR: 2.05, p<10^−4^, z statistic: 4.17) than in those who could be diagnosed with GDM according to only one of the two criteria (Fig 1).

Odds ratios were calculated under the dominant model. Higher OR values indicate the significance of genetic predisposition in those pregnant women who meet both m99’WHO/ IADPSG GDM diagnostic criteria compared to all the other participating pregnant individuals.

#### *CDKAL1* rs7754840 and *GCK* rs799884 risk polymorphisms in GDM

Significant associations were found with the intron variant (rs7754840) of the CDK5 regulatory subunit associated protein 1 like 1 (*CDKAL1*) gene and with the rs1799884 intron variant of the Glucokinase (*GCK*) gene after adjustment to maternal age and pre-pregnancy BMI covariates under the dominant model (OR rs7754840 IADPSG: 1.51, p = 0.016 and OR rs1799884 IADPSG / m99’WHO 1.4 / 1.56, p = 0.04/0.006). Results (ORs, MAFs and adjusted p-values) according to the different diagnostic criteria are indicated in Table 2.

#### Protective polymorphisms, including a novel association signal with GDM

The minor alleles of two common gene variants reduced the risk of developing GDM. A protective common polymorphism was identified as the rs7578326 SNP in the *LOC646736*/*IRS1* region (OR for *G* allele [IADPSG/m99’ WHO]: 0.62/0,60, p = 0.001/0.006) under the dominant model and adjusting to age and pre-pregnancy BMI. Carrying a *T* allele of rs13266634 *C/T* of the *SLC30A8* gene significantly reduced the risk of developing GDM under both diagnostic criteria under the additive model (OR [IADPSG/m99’ WHO]: 0.74/0.71, p = 0.05/0.02) (Table 2).

A causative chain (when the association is not significant after age and BMI adjustments only after age adjustment and the SNP associated effect on GDM develops via BMI) might potentially be existing for a few gene variants (HNF1 homeobox B—*HNF1B* [*TCF2*] -rs4430796, -rs7501939, clock circadian regulator—*CLOCK* -rs6832769,—“S2 Table”), however it needs confirmation with larger sample sizes and we may not yet report these associations as clinically meaningful causative chains due to the diminutive p-value differences after the two adjustments.

### Association between *MTNR1B*, *GCK*, *TCF7L2*, *SLC30A8*, *LOC646736/IRS1* gene variants and glycemic traits

#### Fasting plasma glucose levels

*MTNR1B* rs10830963 was the most significantly associated SNP with the fasting plasma glucose levels (FPG) (mean effect size: 0.21 mmol/L increase, p<5x10^-4^). The *A* allele of the rs1799884 polymorphism of the *GCK* gene was also significantly associated with increased FPG levels (mean effect size 0.13mmol/L increase, p = 0.025). All effect sizes are reported in Table 3 after adjustment to pre-pregnancy BMI and age covariates.

**Table 3:** Association of common gene variants with fasting and 120 minute plasma glucose values at oral glucose tolerance test (OGTT) in pregnant population.

SNP—(major/ minor allele)	Reported Gene (Symbol)	SNP locus (chromosome: base) °	Functional class	MAF in 1000 Genomes in European population	Effect size* on the FPG levels at routine OGTT (24-28^th^ gw) in mmol/L (Confidence Interval**)	Effect size* on the 120 minute postchallenge PG levels at routine OGTT (24-28^th^ gw) in mmol/L (Confidence Interval**)	Model	p (adjusted to BMI and age—FPG / 120' PG)
rs10830963 *(C/G)*	*MTNR1B*	11:92975544	intron variant	0.29	**0.205** (0.1065–0.301)	**0.605** (0.321–0.885)	D	<0.0005/ 0.0005
rs7903146 *(C/T)*	*TCF7L2*	10:112998590	intron variant	0.32	NS	**0.25** (0.014–0.46)	A	NS / 0.027
rs1799884 *(G/A)*	*GCK*	7:44189469	upstream variant	0.18	**0.13** (0.02–0.24)	NS	D	0.025/ NS
rs7578326 *(A/G)*	*lncRNA class RNA gene in the LOC646736/IRS1 region*	2:227020653	intergenic variant	0.35	NS	**-0.45** (-0.75 – -0.14)	D	NS/0.0025
rs13266634	*SLC30A8*	8: 117172544	intron variant. missense	0.28	NS	**-0.26** (-0.48 – 0.03)	A	NS / 0.02

The p-values were adjusted to age and pre-pregnancy body mass index (BMI).

* Effect size of different single nucleotide polymorphisms (SNPs) on FPG/120min and post challenge

PG values were calculated according to the genetic model applied.

Dominant model („D”): effect per carrying the reported minor allele.

Additive model („A”): effect per minor allele.

** Confidence intervals were calculated using bootstrap method.

° To indicate the chromosomal position the NCBI SNP database (release: 107.) was used.

#### Two hour plasma glucose levels at OGTT

The *MTNR1B* rs10830963 was the most significantly associated SNP with the 2 hour PG levels (mean effect size 0.605 mmol/L increase, p = 5x10^-4^) after adjustments to pre-pregnancy BMI and age covariates and this remained significant even when the adjusted p-value was further corrected to reduce FDR using the Benjamini-Hochberg method (p = 0.044). In addition we found an association between the rs7903146 polymorphism of the transcription factor 7-like 2 (*TCF7L2*) gene and 2 hour PG values (mean effect size per *T* allele 0.25mmol/L increase, p = 0.027) after adjustment for pre-pregnancy BMI and age. The rs7578326 SNP in the *LOC646736*/*IRS1* region was associated with decreased (effect size: -0.45 mmol/L, p = 0.0025) 2 hour plasma glucose levels, such as the rs13266634 of the *SLC30A8* gene (effect size per *T* allele: -0.26mmol/L, p = 0.02) (Table 3).

### Pre-pregnancy body mass index

All assessed SNPs of the Melanocortin 4 receptor (*MC4R*, rs10871777, rs571312, rs17782313) were associated with higher pre-pregnancy BMI values at significant level after adjustment to age and using the Benjamini-Hochberg FDR correction method. The LDL receptor related protein 1B (*LRP1B*, rs2890652), Potassium voltage-gated channel subfamily J member 11 (*KCNJ11*, rs5219), CDK5 regulatory subunit associated protein 1 like 1 (*CDKAL1*, rs7754840) and Fat mass and obesity (*FTO*, rs11642841) gene polymorphisms were associated with pre-pregnancy BMI at suggestive levels (Table 4).

**Table 4:** Association of common gene variants with pre-pregnancy body mass index.

SNP—(major/ minor allele)	Reported Gene (Symbol)	SNP locus (chromosome: base) °	Gene function	Functional class	MAF in 1000 Genomes in European population	Genetic model	Effect size	p (adjusted to age)
rs10871777 *(A/G)*	*MC4R*	18:60184530	Membrane-bound receptor and member of the melanocortin receptor family. Defects in this gene are a cause of autosomal dominant obesity.	intergenic variant	0.25	A	1.083	0.0019
rs571312 *(C/A)*	*MC4R*	18:60172536		intron variant	0.24	A	1.123	0.0019
rs177823*13 (T/C)*	*MC4R*	18:60183864		intergenic variant	0.24	A	1.103	0.0018
rs2890652 *(T/C)*	*LRP1B*	2:142202362	Cell surface proteins that bind and internalize ligands in the process of receptor-mediated endocytosis.	intergenic variant	0.17	A	-1.125	0.006
rs5215 *(T/C)*	*KCNJ11*	11:17387083	Inward-rectifier type potassium channel. Associated with the sulfonylurea receptor SUR. Mutations in this gene are a cause of different types of diabetes.	missense, nc transcript variant	0.35	A	0.604	0.048
rs5219 *(C/T)*	*KCNJ11*	11:17388025		missense, nc transcript variant	0.35	A	0.696	0.022
rs7754840 *(G/C)*	*CDKAL1*	6:20661019	Member of the methylthiotransferase family. Gene function is unknown. Gene variants associated with susceptibility to type 2 diabetes.	intron variant	0.32	D	1.157	0.007
rs7756992 *(A/G)*	*CDKAL1*	6:20679478		intron variant	0.28	D	0.923	0.033
rs4712526 *(T/A)*	*CDKAL1*	6:20662804		intron variant	0.32	D	1.008	0.018
rs1164284*1 (C/A)*	*FTO*	16:53811575	Exact function is unknown. Association with body mass index, obesity risk, and type 2 diabetes.	intron variant	0.41	A	0.529	0.07

*MC4R*: Melanocortin 4 Receptor

*LRP1B* Low Density Lipoprotein Receptor-Related Protein 1B

*KCNJ11* Potassium Channel, Inwardly Rectifying Subfamily J, Member 11

*CDKAL1*:CDK5 Regulatory Subunit Associated Protein 1-Like 1

*FTO*: Fat Mass and Obesity-Associated Protein.

All p values were adjusted to age.

° To indicate the chromosomal position the NCBI SNP database (release: 107.) was used

D = dominant genetic model

A = additive genetic model

## Discussion

We conducted a case-control genetic association study in a Caucasian population with 77 gene variants that were either earlier reported to be associated with the GDM or with T2D due to that GDM and T2D share similar genetic susceptibility background [18]. In addition a few other gene variants (SNPs) with potential contribution to an important metabolic condition were also assessed to detect associations with GDM or other related traits.

We found that the *MTNR1B* rs10830963/*G* allele had the most robust association with GDM and also with glycemic traits, including both the FPG and the post-challenge (2 h) PG values at 75g OGTT.

In a recent meta-analysis on GDM genetics the authors concluded that pooled OR for the *MTNR1B* rs10830963 risk variant was lower in Asians (1.23) compared to Caucasians (1.49). Authors found that only in studies which included subjects with mean pre-pregnancy BMI higher than 25 kg/m^2^, but not with lower mean pre-pregnancy BMI values the *MTNR1B* rs10830963 risk variant was significantly associated with GDM [18]. Furthermore, in a recent study with early intervention (diet, lifestyle intervention from the 13^th^ gw) applied in high GDM risk individuals (BMI>30kg/m^2^) only non-carriers of the risk allele *G* benefited from the 3 months intervention assessed by PG values at routine OGTT suggesting that *MTNR1B* rs10830963 variant could modify the efficacy of lifestyle interventions [35]. We found that the rs10830963 *MTNR1B* gene variant is characterized with ORs between 1.84 and 1.64 (dominant model), depending on the diagnostic criteria (IADPSG / m99’WHO) applied. Corroborating reports of higher OR values for rs10830963/*G MTNR1B* found in Caucasians compared to Asians are in accordance with these higher OR values we report here due to that our case cohort was with a mean pre pregnancy BMI higher than 25kg/m^2^ (both in Austria and Hungary) [9, 11, 14, 15, 17, 36, 37].

Accordingly, although several studies found that the rs10830963 *MTNR1B* variant was not associated or only in subgroups associated with fasting and 2 hour glycemic traits, we consistently found associations both with the fasting and post-challenge values with mean 0.21 and 0.61 mmol/L increases in PG levels at OGTT under the dominant model, respectively [11, 12, 14, 15].

Functionally it is important that the *MTNR1B* rs10830963 risk *G* allele is predicted to create a recognition motif that matches the consensus sequences of neuronal differentiation 1 (NEUROD1) and other transcription factors. This was suggested to be consistent with that the risk *G* allele promotes islet MTNR1B expression [38] and the risk *G* allele also increases FOXA2-bound enhancer activity in islet- and liver-derived cells.

The *MTNR1B* rs10830963 intron gene variant seems to be a credible causal gene variant which is the driving association signal in contrast to other candidate SNPs (i.e. rs10830962) that may be retrospectively interpreted as lead gene variants [11, 38].

*MTNR1B* gene encodes one of two high affinity forms of a receptor for melatonin, a pineal gland hormone that regulates glucose metabolism by affecting circadian insulin secretion. *MTNR1B* variants were associated with FPG values [25] and defective *MTNR1B* G-protein-coupled receptor signaling on human beta cells decreased glucose sensitivity and impaired insulin secretion [39]. The rs10830963 was associated with impaired first-phase insulin secretion and decreased Homeostasis Model Assessment—Beta (HOMA-B) even in GDM populations [9, 40].

In a GWAS performed using over 2 million (imputed) initial gene variants Korean authors reported the rs7754840 variant of *CDKAL1* and also the rs10830963 variant of *MTNR1B* as risk polymorphisms in GDM [11]. In our study on a Caucasian population however, *CDKAL1* rs7754840 gene variant was only associated with GDM at a suggestive level. In contrast, the minor, *G* allele of the rs7578326 SNP in the *LOC646736/IRS1* region is first reported here to decrease the risk to develop GDM and to be associated with decreased fasting and 2 hour plasma glucose levels. This protective effect of the minor, G allele is in concordance with prior reports that identified the major, A allele as the risk allele for T2D in a large-scale association analysis [27]. The *IRS1* gene is localized over 500kb downstream of the *LOC646736* genomic region harboring the rs7578326 SNP and the genomic distance of linkage is typically less than 200kb. The *LOC646736* locus and its relation to insulin resistance was characterized and authors suggested clear genotype effects on insulin signaling in skeletal muscle, however did not argue for causality of *IRS1*. Additional pathology might be possible, either via the translation of *LOC646736* transcripts, however due to the lack of putative protein expression identification in human tissues, it is possible that LOC646736 transcripts are linc (long intergenic non-coding) RNAs that alter insulin signal transduction by either cis or trans regulation [41]. These mechanisms may potentially explain the protective effect of the (minor, *G*) variant of the rs7578326 in the *LOC646736* region we first describe in association with GDM development and also with glycemic traits during pregnancy.

The other protective minor (*T*) variant identified in this analysis, the rs13266634 (*C/T*) affects the amino acid residue 325 at the C-terminal of the zinc transporter 8 (*ZnT8*, *SLC30A8)*, which influences zinc homeostasis. In line with prior [11] reports in Asian population our findings confirm the role of this *SLC30A8* variant in Caucasian populations in GDM development. Susceptibility likely evolves via the altered regulation of zinc homeostasis and related changes in insulin production and beta cell function.

The other associations we found with glycemic traits such as the common rs1799884 promoter variant of *GCK* (with increased FPG levels) and the rs7903146 variant of the *TCF7L2* gene (with increased post-challenge PG levels) were reported in prior GDM genetic studies [13, 42, 43].

Regarding the overall architecture of genetic susceptibility of GDM, it should be recognized that many of the gene variants we report here (*MTNR1B*, *CDKAL1*, *SLC30A8*, *TCF7L2* and potentially exerts similar effect) were reported to be associated with a reduced first phase insulin secretion, while the second-phase insulin secretion remained intact [34, 44–47]. In contrast to the above set of gene variants the rs7578326 SNP in the *LOC646736/IRS 1* region clearly affected insulin sensitivity in previous studies [28, 41].

This genetic architecture of GDM predisposition might be especially relevant nowadays, when clinical risk factors, such as higher pre-pregnancy BMI and age [3, 4] at pregnancy become more prevalent resulting in increased insulin resistance and a need for a compensatory increase in beta cell response and the latter might be substantially limited in genetically susceptible individuals.

According to our findings (Fig 1) the most robust association of the causal *MTNR1B* variant rs10830963, is more preponderant in those pregnant individuals who meet both the modified 99’ WHO and the IADPSG diagnostic criteria. In contrast it appears to have a more limited role in determining the odds of disease development in those who could be diagnosed as GDM only according to one of the above diagnostic recommendations, but not by the other. This observation, the “clustering of genetic risk” might points towards precision medicine. Individuals with a susceptible genetic background at risk for developing GDM could potentially benefit from earlier diagnosis and treatment than the currently applied general routine testing (OGTT between the 24-28th gestational week). On the other hand, those who might be diagnosed only according to one of the diagnostic recommendations, the role of lifestyle modifications, age at delivery and related epigenetics may be more substantial factors in determining the odds of disease development (Fig 1).

Genetic markers could possibly also have everyday clinical utility due to that the accuracy of methods predicting GDM and the identification of individuals who are at high risk of developing GDM with pre-pregnancy tests are still inadequate. The sensitivity of clinical risk factor based GDM predictions (conventional risk factors: age, BMI, ethnicity, DM family history, obstetric history—or novel approaches: circulating adiponectin) remains lower than 70% [48, 49].

A limitation of our study is that the sample size allows only the identification of the most robust genetic associations with GDM development (i.e.: OR>1.4) or related traits and did not allow to detect other genetic susceptibility factors that may have a smaller, but still significant effects on disease development or glycemic traits. These data would need confirmation a in a well-powered replication follow-up study.

## Supporting Information

S1 Table List of the 77 SNPs assessed and their reported major function and disease / pathology association. (PDF)

S2 Table **Panel—A. Odds Ratios and Standardized Effect Sizes for each gene variant (SNP) assessed in the study after adjustment by age and BMI.** Data are presented for the two GDM diagnostic criterion (m'99 WHO and IADPSG) systems applied in the study both under the dominant and the additive genetic models. The SNPs identified in this study with GDM as a binary trait are indicated in bold. (See also Table 2 for details). **Panel—B Odds Ratios and Standardized Effect Sizes for each gene variant (SNP) assessed in the Study.** Data are presented for the two GDM diagnostic criterion (m'99 WHO and IADPSG) systems applied in the study both under the dominant and the additive genetic models and adjusted by age only.
(PDF)

S3 Table Minor Allele Frequencies (MAF) in the case-control study populations according to the two GDM diagnostic criteria applied and in the European general population. (PDF)

S4 Table Chi Squared test results for the assessment of the deviation from the Hardy-Weinberg Equilibrium (HWE) genotype distributions. (PDF)

S5 Table The re-calculated mean clinical data of the Austrian and Hungarian pregnant populations. (PDF)
