# Supplementary material for: Association Study with 77 SNPs Confirms the Robust Role for the rs10830963/G of MTNR1B Variant and Identifies Two Novel Associations in Gestational Diabetes Mellitus Development
Source: PLoS One. 2017 Jan 10;12(1):e0169781. doi: 10.1371/journal.pone.0169781 (PMC5224877; doi:10.1371/journal.pone.0169781)
Supplement: S1 Table — (PDF) [file pone.0169781.s001.pdf]

Remark on results	SNP (common variant) ID	Reported Gene (HGNC Symbol)	Reported relevant disease/metabolic/antropometric association trait in the NHGRI-EBI GWAS Catalog*	Relavant reported gene function (based on[#]) or biochemical or other system/pathway involvement from other studies if no GWAS found
	rs12779790	*CDC123, CAMK1D*	T2DM	
	rs4712526	*CDKAL1*	NA	Member of the methylthiotransferase family, other polymorphisms of this gene associated with susceptibilty to T2DM and GDM
*Identified association with GDM binary trait in this sutdy. For more detailed description see Table2 and discussion.	rs7754840*	*CDKAL1*	T2DM, GDM	
	rs7756992	*CDKAL1*	T2DM	
	rs10811661	*CDKN2A/2B*	Fasting plasma glucose, Fasting glucose-related traits (interaction with BMI), T2DM	
	rs1552224	*CENTD2*	T2DM	
	rs5945326	*DUSP9*	T2DM	
	rs7608798	*DPP4*	NA	DPP4 is involved in the regualtion of the incretin system (with enzymatic cleavage of GLP-1 and GIP)
	rs11642841	*FTO*	T2DM, Glycemic trait	
	rs9939609	*FTO*	T2DM, BMI	
*Identified association with GDM binary trait in this sutdy. For more detailed description see Table2 and discussion.	rs1799884	*GCK*	Glycated hemoglobin levels, Glycemic traits	
	rs1260326	*GCKR*	Triglycerides, Cholesterol total, Blood metabolite levels	
	rs780094	*GCKR*	Fasting plasma glucose, Height, Urate levels in obese individuals	
	rs10423928	*GIPR*	Obesity, Two-hour glucose challenge	
	rs1111875	*HHEX/IDE*	T2DM	
	rs5015480	*HHEX/IDE*	T2DM	
	rs11190604	*HIF1AN*	Palmitoleic acid (16:1n-7) plasma levels	
	rs1169288	*HNF1A*	Cholesterol, total, LDL, Liver enzyme levels (gamma gluatamyl transferase)	
	rs1800574	*HNF1A*	NA	Defects in this gene are a cause of maturity onset diabetes of the young type 3 (MODY3)
	rs2464196	*HNF1A*	NA	Defects in this gene are a cause of maturity onset diabetes of the young type 3 (MODY3)
	rs7310409	*HNF1A*	Liver enzyme levels (gamma-glutamyl transferase)	
	rs7957197	*HNF1A*	T2DM	
	rs4430796	*HNF1B*	T2DM	
	rs757210	*HNF1B*	Nonmetabolic trait	
	rs4844880	*HSD11B1*	NA	Catalyzes the conversion of the stress hormone cortisol to the inactive metabolite cortisone, this a variant associated with obesity and IR in children
	rs35767	*IGF1*	Fasting insulin-related traits	
	rs2871865	*IGF1R*	Human height	
	rs3741205	*IGF2*	NA	Insulin-like growth factor system. Encodes a member of the insulin family of polypeptide growth factors, which are involved in development and growth.
	rs1470579	*IGF2BP2*	T2DM, GDM, Fasting glucose-related traits (interaction with BMI)	
	rs4402960	*IGF2BP2*	T2DM	
	rs720390	*IGF2BP2*	Human height	
*Identified association with gylcemic trait in this sutdy. For more detailed description see Table3 and discussion.	rs12534093	*IGF2BP3*	Human height, Infant length	
	rs8191754	*IGF2R*	NA	Insulin-like growth factor system. This gene encodes a receptor for IGF2.
	rs1065780	*IGFBP1*	NA	Insulin-like growth factor system. Regulates bioavailability of IGFs.
	rs9341105	*IGFBP2*	NA	Insulin-like growth factor system. Regulates bioavailability of IGFs and also has IGF independent effects (via RGD and HBD domains).
	rs1143634	*IL1B*	NA	
	rs891088	*INSR*	Obesity-related traits, waist circumference, body fat distribution, body mass index , human height	
	rs10738760	*VLDLR/KCNV2*	Vascular endothelial growth factor levels	
	rs5215	*KCNJ11*	T2DM	
	rs5219	*KCNJ11*	T2DM	
	rs231362	*KCNQ1*	T2DM	
*Identified association with GDM binary trait and glycemic traits in this sutdy. For more detailed description see Table 2-3 and discussion.	rs7578326	*lncRNA class RNA gene in the LOC646736/IRS1 region*	T2DM	
	rs2890652	*LRP1B*	BMI	
	rs571312	*MC4R*	BMI, BMI (age interaction)	
	rs10871777	*MC4R*	Obesity, Hepatic lipid content in extreme obesity	
	rs17782313	*MC4R*	Obesity, BMI, human height	
*Identified association with GDM binary trait and glycemic traits in this sutdy. For more detailed description see Table 2-3 and discussion.	rs10830963	*MTNR1B*	T2DM, GDM, Glucose homeostasis traits, Fasting plasma glucose, Fasting glucose-related traits (interaction with BMI)	
	rs41423247	*NR3C1*	NA	Glucocorticoid receptor gene (NR3C1) polymorphisms are associated with reduced first-phase glucose-stimulated insulin secretion and disposition index in women. NR3C1 polymorphisms are markers for susceptibility to the metabolic syndrome.
	rs6198	*NR3C1*	NA	Glucocorticoid receptor gene (NR3C1) polymorphisms are associated with reduced first-phase glucose-stimulated insulin secretion and disposition index in women. NR3C1 polymorphisms are markers for susceptibility to the metabolic syndrome.
	rs9551419	*PDX1*	NA	Encodes a transcriptional activator of insulin, somatostatin, glucokinase, islet amyloid polypeptide, and GLUT2. Involved in the early development of the pancreas and in glucose-dependent regulation of insulin gene expression. Defects are a cause of pancreatic agenesis, leading to early-onset DM and MODY4
	rs738409	*PNPLA3*	Nonalcoholic fatty liver disease, Liver enzyme levels (alanine transaminase)	
	rs1801282	*PPARG*	T2DM, Fasting insulin-related traits (interaction with BMI)	
	rs11920090	*SLC2A2*	Fasting glucose-related traits, Fasting glucose-related traits (interaction with BMI)	
	rs4846567	*SLC30A10*	Waist-hip ratio	
*Identified association with GDM in this sutdy. For more detailed description see Table2 and discussion.	rs13266634	*SLC30A8*	T2DM, Glycated hemoglobin levels	
	rs1227929	*SLC4A10*	NA	This gene is classified as a member A10 of the SLC4 family of transmembrane solute carriers.
	rs4973768	*SLC4A7*	NA	Solute carrier family 4 member 7 interacts with Cystic fibrosis transmembrane conductance regulator (CFTR) and polymorphisms associated with cardiovascular disease risk
	rs7501939	*HNF1B*	NA	Regulates development of the embryonic pancreas. Mutations in this gene result in diabetes syndrome (MODY5) and noninsulin-dependent diabetes mellitus
	rs12243326	*TCF7L2*	Two-hour glucose challenge	
	rs12255372	*TCF7L2*	NA	This gene encodes a transcription factor that plays a key role in the Wnt signaling pathway. The protein has been implicated in blood glucose homeostasis. Genetic variants of this gene are associated with increased risk of type 2 diabetes. Polymorphisms in this gene influences the insulin release of Beta cells upon incretin hormon stimulation.
*Identified association with gylcemic trait in this sutdy. For more detailed description see Table3 and discussion.	rs7903146	*TCF7L2*	T2DM, BMI	
	rs6884205	*TGFB2*	NA	This gene encodes a member of the transforming growth factor beta (TGFB) family of cytokines, contributes to regulation of Treg cells that influences T2DM pathogenesis.
	rs2867125	*TMEM18*	BMI	
	rs12463617	*TMEM18*	BMI, Obesity (early onset extreme)	
	rs6905288	*VEGFA*	Waist-hip ratio, CHD	
	rs6921438	*VEGFA*	NA	This gene is a member of the PDGF/VEGF growth factor family. VEGF levels may contribute to GDM pathology.
	rs10010131	*WFS1*	NA	This gene encodes a transmembrane protein. Mutations are associated with Wolfram syndrome, DIDMOAD (Diabetes Insipidus, Diabetes Mellitus, Optic Atrophy, and Deafness). Common variants are associated with glycemic traits and T2DM susceptibility. Polymorphisms in this gene influences the insulin release of Beta cells upon incretin hormon stimulation.
	rs1801214	*WFS1*	T2DM	
	rs4689388	*WFS1*	T2DM, Glycemic traits	
	rs734312	*WFS1*	NA	see above
	rs6993770	*ZFPM2*	Vascular endothelial growth factor levels	
	rs72865282	*AC092841.1 (miRNA)*	NA	Target candidate gene is a cytoplasmic viral RNA sensor linked to other diabetes forms
	rs11708067	*ADCY5*	NA	Single nucleotide polymorphisms in this gene may be associated with low birth weight and T2DM.
	rs7950226	*ARNTL*	NA	The protein encoded forms a heterodimer with CLOCK. Required for beta cell compensatory expansion, survival and metabolic adaptation to diet-induced obesity in diabetes models.
	rs6832769	*CLOCK*	Behavior measurement	Circadian clock gene expression is impaired in gestational diabetes mellitus.
	rs900145	*ARNTL*	Body fat distribution, age at menarche	
	rs2286615	*BAD*	NA	The protein encoded is a member of the BCL-2 family - known to be regulators of programmed cell death. GWAS recently identified BCL2 as novel Insulin sensitivity locus.

* https://www.ebi.ac.uk/gwas/ - last accessed: 07/24/2016

[#] http://www.ncbi.nlm.nih.gov/gene - last accessed: 07/24/2016

Supplementary Table 1

List of the 77 SNPs assessed and their reported major function and disease / pathology aoosciation.
