# Supplementary material for: Association Study with 77 SNPs Confirms the Robust Role for the rs10830963/G of MTNR1B Variant and Identifies Two Novel Associations in Gestational Diabetes Mellitus Development
Source: PLoS One. 2017 Jan 10;12(1):e0169781. doi: 10.1371/journal.pone.0169781 (PMC5224877; doi:10.1371/journal.pone.0169781)
Supplement: S2 Table — Panel—A. Odds Ratios and Standardized Effect Sizes for each gene variant (SNP) assessed in the study after adjustment by age and BMI. Data are presented for the two GDM diagnostic criterion (m'99 WHO and IADPSG) systems applied in the study both under the dominant and the additive genetic models. The SNPs identified in this study with GDM as a binary trait are indicated in bold. (See also Table 2 for details). Panel—B Odds Ratios and Standardized Effect Sizes for each gene variant (SNP) assessed in the Study. Data are presented for the two GDM diagnostic criterion (m'99 WHO and IADPSG) systems applied in the study both under the dominant and the additive genetic models and adjusted by age only. (PDF) [file pone.0169781.s002.pdf]

SNP ID	Reported Gene Name (HGNC Abbreviation)	Dominant genetic model						Additive genetic model					
		OR m'99 WHO	p m'99 WHO	OR IADPSG	p IADPSG	Standardized effect size m99' WHO	Standardized effect size m99' WHO	OR m'99 WHO	p m'99 WHO	OR IADPSG	p IADPSG	Standardized effect size m99' WHO	Standardized effect size IADPSG
rs10010131	*WFS1*	0,85	0,412	1,09	0,6567	-0,080	0,044	0,91	0,494	1,05	0,747	-0,066	0,031
rs10423928	*GIPR*	0,88	0,477	1,02	0,8945	-0,064	0,012	0,94	0,682	0,99	0,933	-0,035	-0,007
rs1065780	*IGFBP1*	0,71	0,063	0,80	0,2233	-0,169	-0,110	0,72	0,018	0,86	0,257	-0,220	-0,104
rs10738760	*VLDLR/KCNV2*	1,04	0,838	0,85	0,4080	0,018	-0,072	1,06	0,612	0,98	0,867	0,044	-0,015
rs10811661	*CDKN2A/2B*	0,99	0,975	0,91	0,6178	-0,003	-0,045	0,96	0,821	0,80	0,198	-0,020	-0,118
**rs10830963**	***MTNR1B***	**1,64**	**0,006**	**1,85**	**0,0008**	**0,249**	**0,308**	**1,39**	**0,012**	**1,48**	**0,0032**	**0,222**	**0,264**
rs10871777	*MC4R*	1,17	0,382	1,05	0,7996	0,078	0,023	1,11	0,463	0,98	0,897	0,065	-0,012
rs1111875	*HHEX/IDE*	0,85	0,383	0,84	0,3380	-0,077	-0,085	0,82	0,135	0,83	0,141	-0,133	-0,131
rs11190604	*HIF1AN*	0,97	0,878	1,23	0,2719	-0,014	0,098	0,98	0,918	1,13	0,405	-0,009	0,070
rs1143634	*IL1B*	1,20	0,307	1,10	0,5920	0,092	0,048	1,14	0,379	1,02	0,875	0,080	0,014
rs11642841	*FTO*	0,58	0,004**	0,63	0,0139**	-0,256	-0,219	0,78	0,046	0,74	0,018	-0,181	-0,215
rs1169288	*HNF1A*	1,19	0,359	0,78	0,1898	0,084	-0,120	1,16	0,241	0,85	0,228	0,106	-0,111
rs11708067	*ADCY5*	0,90	0,652	1,31	0,2338	-0,049	0,129	0,89	0,537	1,28	0,189	-0,069	0,142
rs11920090	*SLC2A2*	0,82	0,374	0,77	0,2532	-0,084	-0,109	0,75	0,146	0,74	0,144	-0,144	-0,146
rs12243326	*TCF7L2*	1,23	0,259	0,95	0,7680	0,102	-0,027	1,24	0,127	1,08	0,604	0,135	0,046
rs12255372	*TCF7L2*	1,31	0,132	0,93	0,6675	0,136	-0,039	1,31	0,049	1,09	0,549	0,174	0,054
rs1227929	*SLC4A10*	0,74	0,100	0,84	0,3415	-0,148	-0,086	0,84	0,162	0,90	0,371	-0,128	-0,082
rs12463617	*TMEM18*	1,16	0,430	1,46	0,0497	0,071	0,176	1,05	0,749	1,30	0,107	0,028	0,142
rs12534093	*IGF2BP3*	1,13	0,504	1,07	0,7056	0,060	0,034	1,23	0,178	1,13	0,429	0,120	0,071
rs1260326	*GCKR*	0,94	0,745	0,89	0,5745	-0,030	-0,051	0,97	0,841	0,86	0,257	-0,018	-0,105
rs12779790	*CDC123, CAMK1D*	0,94	0,753	0,75	0,1600	-0,029	-0,133	0,97	0,884	0,76	0,137	-0,013	-0,141
**rs13266634**	***SLC30A8***	**0,74**	**0,101**	**0,71**	**0,0583**	**-0,149**	**-0,173**	**0,71**	**0,021**	**0,75**	**0,048**	**-0,221**	**-0,188**
rs1470579	*IGF2BP2*	0,85	0,377	1,04	0,8271	-0,079	0,020	0,91	0,509	1,03	0,804	-0,060	0,022
rs1552224	*CENTD2*	0,92	0,694	1,09	0,6788	-0,037	0,038	0,93	0,700	1,11	0,588	-0,037	0,052
rs17782313	*MC4R*	1,16	0,419	1,03	0,8574	0,073	0,016	1,11	0,478	0,98	0,871	0,064	-0,015
**rs1799884**	***GCK***	**1,67**	**0,008**	**1,44**	**0,0611**	**0,233**	**0,164**	**1,56**	**0,006**	**1,40**	**0,039**	**0,229**	**0,173**
rs1800574	*HNF1A*	1,32	0,468	0,99	0,9795	0,057	-0,002	1,46	0,300	1,13	0,757	0,081	0,025
rs1801214	*WFS1*	0,89	0,523	1,13	0,5008	-0,058	0,062	0,92	0,530	1,06	0,672	-0,055	0,037
rs1801282	*PPARG*	0,82	0,345	0,83	0,3787	-0,086	-0,080	0,84	0,340	0,87	0,457	-0,084	-0,065
rs2286615	*BAD*	0,95	0,819	0,93	0,7592	-0,022	-0,030	0,88	0,544	0,87	0,503	-0,062	-0,069
rs231362	*KCNQ1*	0,98	0,908	0,70	0,0701	-0,011	-0,163	0,98	0,867	0,84	0,158	-0,015	-0,127
rs2464196	*HNF1A*	1,03	0,884	0,90	0,6350	0,016	-0,053	1,07	0,694	1,05	0,784	0,044	0,031
rs2867125	*TMEM18*	1,09	0,651	1,31	0,1687	0,041	0,124	1,03	0,866	1,25	0,191	0,015	0,118
rs2871865	*IGF1R*	0,87	0,542	1,02	0,9407	-0,056	0,007	0,89	0,578	0,97	0,886	-0,052	-0,013
**rs2890652**	***LRP1B***	**1,52**	**0,027**	**1,47**	**0,0425**	**0,196**	**0,180**	**1,38**	**0,055**	**1,42**	**0,037**	**0,168**	**0,183**
rs35767	*IGF1*	0,73	0,116	1,21	0,3191	-0,144	0,089	0,80	0,192	1,24	0,190	-0,117	0,114
rs3741205	*IGF2*	1,02	0,925	0,79	0,3101	0,010	-0,117	0,93	0,607	0,79	0,107	-0,056	-0,182
rs41423247	*NR3C1*	0,84	0,320	0,70	0,0492	-0,089	-0,177	0,80	0,111	0,75	0,036	-0,144	-0,192
rs4402960	*IGF2BP2*	0,86	0,393	1,11	0,5597	-0,077	0,053	0,91	0,501	1,07	0,609	-0,060	0,045
rs4430796	*HNF1B (TCF2)*	0,65	0,025	0,91	0,6228	-0,194	-0,043	0,79	0,066	0,95	0,667	-0,165	-0,038
rs4689388	*WFS1*	0,87	0,448	1,10	0,6106	-0,067	0,046	0,91	0,449	1,03	0,796	-0,066	0,023
rs4712526	*CDKAL1*	1,22	0,264	1,30	0,1440	0,101	0,133	1,04	0,765	1,10	0,470	0,026	0,064
rs4844880	*HSD11B1*	1,28	0,193	1,41	0,0706	0,114	0,159	1,30	0,097	1,39	0,036	0,144	0,182
rs4846567	*SLC30A10*	0,87	0,452	0,86	0,4337	-0,069	-0,072	0,94	0,710	0,89	0,464	-0,034	-0,069
rs4973768	*SLC4A7*	0,90	0,604	1,01	0,9741	-0,047	0,003	1,02	0,863	0,96	0,741	0,016	-0,031
rs5015480	*HHEX/IDE*	0,90	0,563	0,92	0,6368	-0,051	-0,042	0,85	0,189	0,86	0,246	-0,115	-0,102
rs5215	*KCNJ11*	0,98	0,895	0,80	0,2229	-0,012	-0,111	1,03	0,792	0,92	0,527	0,023	-0,056
rs5219	*KCNJ11*	0,99	0,938	0,81	0,2739	-0,007	-0,101	1,04	0,767	0,93	0,584	0,026	-0,049
rs571312	*MC4R*	1,14	0,471	0,98	0,8986	0,065	-0,012	1,12	0,455	0,93	0,647	0,068	-0,042
rs5945326	*DUSP9*	0,92	0,659	0,95	0,7628	-0,039	-0,027	0,85	0,284	0,93	0,648	-0,112	-0,047
rs6198	*NR3C1*	0,71	0,148	0,85	0,5040	-0,159	-0,072	0,71	0,108	0,89	0,577	-0,183	-0,062
rs6832769	*CLOCK*	1,31	0,142	1,27	0,1890	0,134	0,119	1,30	0,058	1,21	0,159	0,172	0,127
rs6884205	*TGFB2*	0,90	0,564	0,99	0,9400	-0,052	-0,007	0,93	0,670	1,03	0,873	-0,039	0,015
rs6905288	*VEGFA*	0,88	0,490	1,25	0,2548	-0,062	0,104	0,91	0,469	1,05	0,717	-0,066	0,033
rs6921438	*VEGFA*	1,35	0,136	1,30	0,1949	0,138	0,120	1,02	0,889	1,06	0,637	0,013	0,044
rs6993770	*ZFPM2*	0,84	0,328	1,01	0,9468	-0,088	0,006	0,89	0,426	1,00	0,999	-0,073	0,000
rs720390	*IGF2BP2*	0,77	0,184	0,99	0,9681	-0,128	-0,004	0,84	0,245	1,00	0,981	-0,115	-0,002
rs72865282	*AC092841.1 (miRNA)*	0,93	0,771	1,15	0,5767	-0,030	0,057	0,97	0,886	1,17	0,511	-0,015	0,068
rs7310409	*HNF1A*	1,06	0,748	0,86	0,4300	0,029	-0,071	1,13	0,336	0,98	0,882	0,087	-0,013
rs734312	*WFS1*	0,80	0,236	0,95	0,7735	-0,104	-0,026	0,85	0,180	0,97	0,836	-0,118	-0,018
rs738409	*PNPLA3*	0,94	0,735	0,92	0,6592	-0,031	-0,040	0,98	0,879	0,96	0,776	-0,014	-0,027
rs7501939	*HNF1B (TCF2)*	0,69	0,037	0,89	0,5039	-0,185	-0,060	0,79	0,064	0,92	0,540	-0,166	-0,054
rs757210	*HNF1B (TCF2)*	0,67	0,071	0,94	0,7846	-0,199	-0,030	0,79	0,134	0,90	0,526	-0,168	-0,070
**rs7578326**	***lncRNA class RNA gene in the LOC646736/IRS1 region***	**0,61**	**0,006**	**0,56**	**0,0016**	**-0,247**	**-0,285**	**0,71**	**0,014**	**0,66**	**0,002**	**-0,229**	**-0,282**
rs7608798	*DPP4*	1,06	0,785	1,26	0,2523	0,027	0,115	1,09	0,582	1,15	0,339	0,055	0,097
**rs7754840**	***CDKAL1***	**1,25**	**0,213**	**1,34**	**0,1055**	**0,113**	**0,148**	**1,06**	**0,671**	**1,13**	**0,353**	**0,038**	**0,082**
rs7756992	*CDKAL1*	1,09	0,617	1,27	0,1838	0,045	0,120	1,05	0,725	1,15	0,303	0,031	0,091
rs780094	*GCKR*	0,89	0,553	0,87	0,4681	-0,053	-0,066	0,98	0,869	0,88	0,328	-0,015	-0,091
rs7903146	*TCF7L2*	1,32	0,131	1,04	0,8173	0,137	0,021	1,31	0,049	1,19	0,210	0,175	0,112
rs7950226	*ARNTL*	0,64	0,099	1,08	0,7866	-0,202	0,035	0,72	0,081	1,01	0,960	-0,228	0,007
rs7957197	*HNF1A*	0,85	0,404	0,81	0,2757	-0,076	-0,099	0,87	0,424	0,82	0,228	-0,073	-0,111
rs8191754	*IGF2R*	1,22	0,359	1,11	0,6289	0,084	0,045	1,19	0,336	1,11	0,564	0,087	0,053
rs891088	*INSR*	1,37	0,077	1,51	0,0227	0,158	0,204	1,24	0,123	1,36	0,028	0,136	0,194
rs900145	*ARNTL*	1,20	0,319	0,92	0,6507	0,092	-0,042	1,08	0,577	0,85	0,238	0,050	-0,108
rs9341105	*IGFBP2*	1,30	0,150	0,96	0,8078	0,130	-0,022	1,30	0,074	0,99	0,953	0,164	-0,005
rs9551419	*PDX1*	0,75	0,153	0,78	0,2288	-0,129	-0,109	0,77	0,153	0,74	0,112	-0,130	-0,146
rs9939609	*FTO*	0,77	0,170	0,70	0,0585	-0,123	-0,171	0,85	0,207	0,74	0,021**	-0,113	-0,211

Supplementary Table 2.
Panel - A
Odds Ratios and Standardized Effect Sizes for each gene variant (SNP) assessed in the study after adjustment by age and BMI.
Data are presented for the two GDM diagnostic criterion (m'99 WHO and IADPSG) systems applied in the study both under the dominant and the additive genetic models.
The SNPs identified in this study with GDM as a binary trait are indicated in bold. (See also Table 2 for details).

SNP ID	Reported Gene Name (HGNC Abbreviation)	Dominant genetic model				Additive genetic model			
		OR m'99 WHO	p m'99 WHO	OR IADPSG	p IADPSG	OR m'99 WHO	p m'99 WHO	OR IADPSG	p IADPSG
rs10010131	*WFS1*	0,891	0,539	1,100	0,617	0,943	0,660	1,066	0,628
rs10423928	*GIPR*	0,879	0,455	1,022	0,898	0,944	0,673	0,996	0,978
rs1065780	*IGFBP1*	0,781	0,152	0,861	0,379	0,752	0,032	0,876	0,307
rs10738760	*VLDLR/KCNV2*	0,991	0,960	0,817	0,277	1,058	0,624	0,973	0,811
rs10811661	*CDKN2A/2B*	1,022	0,905	0,953	0,795	0,985	0,920	0,848	0,297
rs10830963	*MTNR1B*	1,672	0,003	1,803	0,001	1,414	0,005	1,471	0,002
rs10871777	*MC4R*	1,270	0,165	1,166	0,368	1,198	0,189	1,086	0,549
rs1111875	*HHEX/IDE*	0,841	0,315	0,818	0,240	0,820	0,103	0,817	0,095
rs11190604	*HIF1AN*	1,089	0,625	1,361	0,075	1,074	0,620	1,237	0,131
rs1143634	*IL1B*	1,094	0,601	1,020	0,909	1,107	0,468	1,015	0,917
rs11642841	*FTO*	0,753	0,106	0,814	0,238	0,882	0,285	0,853	0,174
rs1169288	*HNF1A*	1,160	0,407	0,802	0,209	1,162	0,216	0,889	0,333
rs11708067	*ADCY5*	0,953	0,821	1,297	0,220	0,924	0,661	1,263	0,186
rs11920090	*SLC2A2*	0,760	0,192	0,721	0,120	0,685	0,046	0,681	0,042
rs12243326	*TCF7L2*	1,122	0,497	0,900	0,534	1,156	0,268	1,025	0,852
rs12255372	*TCF7L2*	1,224	0,236	0,904	0,552	1,237	0,105	1,049	0,715
rs1227929	*SLC4A10*	0,787	0,162	0,873	0,427	0,852	0,173	0,902	0,372
rs12463617	*TMEM18*	1,074	0,696	1,315	0,130	1,011	0,943	1,228	0,183
rs12534093	*IGF2BP3*	0,962	0,824	0,910	0,590	1,083	0,578	1,000	0,999
rs1260326	*GCKR*	0,915	0,639	0,872	0,462	0,939	0,599	0,842	0,150
rs12779790	*CDC123, CAMK1D*	1,080	0,683	0,871	0,471	1,147	0,397	0,901	0,527
rs13266634	*SLC30A8*	0,758	0,106	0,719	0,054	0,728	0,022	0,759	0,045
rs1470579	*IGF2BP2*	0,893	0,504	1,077	0,661	0,915	0,488	1,026	0,839
rs1552224	*CENTD2*	0,888	0,548	1,033	0,869	0,912	0,612	1,067	0,716
rs17782313	*MC4R*	1,271	0,166	1,167	0,369	1,203	0,187	1,087	0,550
rs1799884	*GCK*	1,437	0,047	1,207	0,303	1,378	0,040	1,212	0,217
rs1800574	*HNF1A*	1,057	0,878	0,801	0,557	1,155	0,678	0,896	0,760
rs1801214	*WFS1*	0,921	0,635	1,140	0,448	0,953	0,692	1,077	0,534
rs1801282	*PPARG*	0,838	0,383	0,820	0,325	0,855	0,380	0,866	0,415
rs2286615	*BAD*	1,000	0,998	0,980	0,924	0,929	0,702	0,912	0,630
rs231362	*KCNQ1*	0,925	0,675	0,709	0,060	0,957	0,708	0,848	0,158
rs2464196	*HNF1A*	1,099	0,658	0,973	0,896	1,077	0,641	1,057	0,732
rs2867125	*TMEM18*	1,006	0,976	1,183	0,362	0,976	0,884	1,169	0,336
rs2871865	*IGF1R*	0,890	0,591	1,039	0,856	0,922	0,694	1,017	0,933
rs2890652	*LRP1B*	1,264	0,191	1,214	0,277	1,181	0,298	1,200	0,250
rs35767	*IGF1*	0,786	0,193	1,198	0,313	0,865	0,355	1,248	0,141
rs3741205	*IGF2*	0,987	0,952	0,757	0,204	0,934	0,616	0,800	0,109
rs41423247	*NR3C1*	0,869	0,407	0,737	0,071	0,800	0,084	0,748	0,024
rs4402960	*IGF2BP2*	0,922	0,633	1,178	0,335	0,933	0,594	1,085	0,522
rs4430796	*HNF1B (TCF2)*	0,642	0,014	0,885	0,496	0,745	0,012	0,879	0,264
rs4689388	*WFS1*	0,905	0,566	1,101	0,579	0,953	0,691	1,069	0,578
rs4712526	*CDKAL1*	1,346	0,084	1,456	0,028	1,107	0,412	1,176	0,183
rs4844880	*HSD11B1*	1,226	0,261	1,354	0,092	1,249	0,140	1,339	0,051
rs4846567	*SLC30A10*	0,901	0,555	0,880	0,464	0,961	0,787	0,904	0,490
rs4973768	*SLC4A7*	0,909	0,617	1,032	0,869	1,019	0,876	0,976	0,841
rs5015480	*HHEX/IDE*	0,889	0,496	0,891	0,498	0,843	0,159	0,853	0,185
rs5215	*KCNJ11*	1,056	0,757	0,900	0,542	1,075	0,549	0,983	0,886
rs5219	*KCNJ11*	1,092	0,619	0,944	0,740	1,100	0,436	1,014	0,908
rs571312	*MC4R*	1,266	0,176	1,099	0,587	1,223	0,156	1,045	0,756
rs5945326	*DUSP9*	0,994	0,970	1,034	0,844	0,929	0,612	1,031	0,830
rs6198	*NR3C1*	0,721	0,149	0,876	0,552	0,724	0,103	0,899	0,578
rs6832769	*CLOCK*	1,439	0,037	1,382	0,061	1,368	0,016	1,258	0,073
rs6884205	*TGFB2*	0,934	0,702	1,035	0,846	0,938	0,681	1,034	0,825
rs6905288	*VEGFA*	0,893	0,533	1,199	0,319	0,917	0,483	1,036	0,773
rs6921438	*VEGFA*	1,208	0,319	1,174	0,392	0,989	0,928	1,033	0,792
rs6993770	*ZFPM2*	0,858	0,369	0,995	0,975	0,922	0,543	0,990	0,939
rs720390	*IGF2BP2*	0,806	0,247	1,033	0,863	0,872	0,325	1,026	0,853
rs72865282	*AC092841.1 (miRNA)*	0,880	0,599	1,062	0,802	0,935	0,761	1,090	0,687
rs7310409	*HNF1A*	1,098	0,609	0,902	0,564	1,117	0,351	0,986	0,903
rs734312	*WFS1*	0,921	0,641	1,064	0,728	0,922	0,486	1,046	0,699
rs738409	*PNPLA3*	0,900	0,548	0,860	0,385	0,931	0,638	0,895	0,462
rs7501939	*HNF1B (TCF2)*	0,664	0,017	0,848	0,334	0,735	0,013	0,854	0,192
rs757210	*HNF1B (TCF2)*	0,658	0,048	0,912	0,665	0,772	0,091	0,885	0,421
rs7578326	*lncRNA class RNA gene in the LOC646736/IRS1 region*	0,670	0,022	0,621	0,006	0,772	0,044	0,720	0,010
rs7608798	*DPP4*	0,953	0,798	1,101	0,610	1,020	0,887	1,069	0,639
rs7754840	*CDKAL1*	1,385	0,057	1,506	0,017	1,132	0,316	1,214	0,113
rs7756992	*CDKAL1*	1,198	0,287	1,398	0,048	1,135	0,317	1,244	0,082
rs780094	*GCKR*	0,886	0,515	0,859	0,409	0,955	0,704	0,867	0,236
rs7903146	*TCF7L2*	1,187	0,317	0,968	0,847	1,204	0,150	1,104	0,443
rs7950226	*ARNTL*	0,685	0,145	1,088	0,755	0,777	0,165	1,032	0,865
rs7957197	*HNF1A*	0,794	0,211	0,765	0,145	0,817	0,206	0,773	0,107
rs8191754	*IGF2R*	1,124	0,564	1,030	0,883	1,103	0,575	1,031	0,863
rs891088	*INSR*	1,310	0,111	1,440	0,030	1,211	0,154	1,330	0,033
rs900145	*ARNTL*	1,180	0,344	0,950	0,765	1,090	0,501	0,901	0,418
rs9341105	*IGFBP2*	1,175	0,351	0,902	0,550	1,219	0,151	0,968	0,812
rs9551419	*PDX1*	0,744	0,120	0,782	0,191	0,756	0,107	0,742	0,084
rs9939609	*FTO*	0,917	0,627	0,844	0,339	0,948	0,651	0,850	0,167

Supplementary Table 2.
Panel - B
Odds Ratios and Standardized Effect Sizes for each gene variant (SNP) assessed in the Study.
Data are presented for the two GDM diagnostic criterion (m'99 WHO and IADPSG) systems applied in the study both under the dominant and the additive genetic models and adjusted by age only.
