# Supplementary material for: Association Study with 77 SNPs Confirms the Robust Role for the rs10830963/G of MTNR1B Variant and Identifies Two Novel Associations in Gestational Diabetes Mellitus Development
Source: PLoS One. 2017 Jan 10;12(1):e0169781. doi: 10.1371/journal.pone.0169781 (PMC5224877; doi:10.1371/journal.pone.0169781)
Supplement: S3 Table — (PDF) [file pone.0169781.s003.pdf]

SNP Id	Reported Gene Name (HGNC Abbreviation)	MAF IADPSG- case	MAF IADPSG- control	MAF m99'WHO- case	MAF m99' WHO- control	Entire Study Population	MAF_EU poplation_(1000 Genomes)	Minor allel	
rs10010131	*WFS1*	0,367	0,359	0,345	0,368	0,362	0,37	A	
rs10423928	*GIPR*	0,257	0,255	0,251	0,257	0,255	0,21	A	
rs1065780	*IGFBP1*	0,327	0,347	0,304	0,356	0,34	0,39	A	
rs10738760	*VLDLR/KCNV2*	0,488	0,492	0,5	0,485	0,491	0,5	A	
rs10811661	*CDKN2A/2B*	0,149	0,173	0,164	0,165	0,165	0,17	C	
**rs10830963**	***MTNR1B***	**0,361**	**0,277**	**0,355**	**0,281**	**0,304**	**0,29**	**G**	
rs10871777	*MC4R*	0,245	0,223	0,259	0,218	0,23	0,25	G	
rs1111875	*HHEX/IDE*	0,326	0,38	0,325	0,38	0,363	0,42	T	
rs11190604	*HIF1AN*	0,244	0,204	0,228	0,212	0,217	0,19	G	
rs1143634	*IL1B*	0,235	0,24	0,248	0,235	0,239	0,25	A	
rs11642841	*FTO*	0,38	0,433	0,385	0,429	0,416	0,41	A	
rs1169288	*HNF1A*	0,373	0,392	0,419	0,37	0,385	0,34	C	
rs11708067	*ADCY5*	0,239	0,191	0,202	0,208	0,207	0,17	G	
rs11920090	*SLC2A2*	0,094	0,128	0,096	0,128	0,117	0,14	A	
rs12243326	*TCF7L2*	0,306	0,289	0,326	0,281	0,295	0,29	C	
rs12255372	*TCF7L2*	0,32	0,3	0,345	0,289	0,306	0,29	T	
rs1227929	*SLC4A10*	0,34	0,368	0,329	0,374	0,359	0,4	T	
rs12463617	*TMEM18*	0,194	0,163	0,175	0,172	0,173	0,17	A	
rs12534093	*IGF2BP3*	0,211	0,204	0,222	0,198	0,206	0,23	A	
rs1260326	*GCKR*	0,438	0,487	0,457	0,478	0,472	0,41	T	
rs12779790	*CDC123, CAMK1D*	0,135	0,154	0,153	0,146	0,148	0,2	G	
**rs13266634**	***SLC30A8***	**0,25**	**0,302**	**0,244**	**0,305**	**0,285**	**0,28**	**T**	
rs1470579	*IGF2BP2*	0,295	0,304	0,278	0,313	0,301	0,3	C	
rs1552224	*CENTD2*	0,134	0,134	0,123	0,14	0,134	0,15	C	
rs17782313	*MC4R*	0,241	0,218	0,255	0,213	0,226	0,24	C	
**rs1799884**	***GCK***	**0,187**	**0,15**	**0,198**	**0,144**	**0,162**	**0,18**	**T**	
rs1800574	*HNF1A*	0,027	0,029	0,033	0,026	0,028	0,03	T	
rs1801214	*WFS1*	0,365	0,349	0,344	0,358	0,354	0,36	C*	* multi-allelic
rs1801282	*PPARG*	0,123	0,132	0,12	0,133	0,129	0,12	G	
rs2286615	*BAD*	0,107	0,129	0,108	0,128	0,122	0,16	A	
rs231362	*KCNQ1*	0,451	0,483	0,475	0,47	0,473	0,5	A	
rs2464196	*HNF1A*	0,322	0,311	0,325	0,31	0,315	0,32	A	
rs2867125	*TMEM18*	0,182	0,159	0,166	0,166	0,166	0,17	T	
rs2871865	*IGF1R*	0,105	0,098	0,1	0,1	0,1	0,13	G	
rs2890652	*LRP1B*	0,188	0,165	0,187	0,165	0,172	0,17	C	
rs35767	*IGF1*	0,21	0,171	0,169	0,19	0,183	0,16	A	
rs3741205	*IGF2*	0,274	0,335	0,302	0,319	0,315	0,3	C	
rs41423247	*NR3C1*	0,263	0,334	0,271	0,329	0,311	0,38	C	
rs4402960	*IGF2BP2*	0,312	0,311	0,291	0,322	0,312	0,3	T	
rs4430796	*HNF1B(TCF2)*	0,442	0,469	0,414	0,48	0,46	0,48	G	
rs4689388	*WFS1*	0,384	0,371	0,363	0,38	0,375	0,38	G	
rs4712526	*CDKAL1*	0,35	0,31	0,342	0,312	0,322	0,32	A	
rs4844880	*HSD11B1*	0,206	0,16	0,2	0,163	0,175	0,16	A	
rs4846567	*SLC30A10*	0,209	0,235	0,214	0,233	0,227	0,29	T	
rs4973768	*SLC4A7*	0,463	0,476	0,472	0,471	0,472	0,48	T	
rs5015480	*HHEX/IDE*	0,341	0,384	0,338	0,385	0,37	0,43	T	
rs5215	*KCNJ11*	0,368	0,377	0,383	0,371	0,374	0,35	C	
rs5219	*KCNJ11*	0,371	0,374	0,385	0,368	0,373	0,35	T	
rs571312	*MC4R*	0,228	0,213	0,246	0,206	0,218	0,24	A	
rs5945326	*DUSP9*	0,225	0,228	0,213	0,232	0,227	0,25	G	
rs6198	*NR3C1*	0,175	0,186	0,156	0,195	0,182	0,17	C	
rs6832769	*CLOCK*	0,36	0,313	0,371	0,31	0,328	0,35	G	
rs6884205	*TGFB2*	0,2	0,197	0,192	0,201	0,198	0,16	T	
rs6905288	*VEGFA*	0,429	0,421	0,41	0,431	0,424	0,4	G	
rs6921438	*VEGFA*	0,442	0,436	0,438	0,439	0,438	0,47	A	
rs6993770	*ZFPM2*	0,289	0,287	0,276	0,293	0,288	0,3	T	
rs720390	*IGF2BP2*	0,348	0,36	0,325	0,37	0,356	0,38	A	
rs72865282	*AC092841.1 (miRNA)*	0,103	0,093	0,093	0,098	0,096	0,1	C	
rs7310409	*HNF1A*	0,44	0,433	0,461	0,423	0,435	0,42	A	
rs734312	*WFS1*	0,417	0,419	0,394	0,43	0,419	0,44	G	
rs738409	*PNPLA3*	0,21	0,224	0,212	0,222	0,22	0,23	G	
rs7501939	*HNF1B(TCF2)*	0,342	0,367	0,318	0,377	0,359	0,39	T	
rs757210	*HNF1B(TCF2)*	0,33	0,363	0,309	0,37	0,352	0,35	T*	multi-allelic
**rs7578326**	***lncRNA class RNA gene in the LOC646736/IRS1 region***	**0,311**	**0,398**	**0,32**	**0,393**	**0,371**	**0,35**	**G**	
rs7608798	*DPP4*	0,359	0,341	0,352	0,344	0,346	0,34	A	
**rs7754840**	***CDKAL1***	**0,355**	**0,307**	**0,344**	**0,31**	**0,322**	**0,32**	**C**	
rs7756992	*CDKAL1*	0,314	0,268	0,3	0,273	0,282	0,28	G	
rs780094	*GCKR*	0,426	0,47	0,442	0,462	0,456	0,41	T	
rs7903146	*TCF7L2*	0,334	0,304	0,349	0,297	0,313	0,32	T	
rs7950226	*ARNTL*	0,447	0,44	0,402	0,463	0,442	0,46	A	
rs7957197	*HNF1A*	0,155	0,188	0,161	0,186	0,178	0,2	A	
rs8191754	*IGF2R*	0,127	0,122	0,133	0,119	0,124	0,15	G	
rs891088	*INSR*	0,301	0,241	0,29	0,246	0,26	0,27	G	
rs900145	*ARNTL*	0,273	0,299	0,304	0,284	0,291	0,28	C	
rs9341105	*IGFBP2*	0,241	0,249	0,273	0,236	0,247	0,28	G	
rs9551419	*PDX1*	0,135	0,167	0,138	0,166	0,157	0,16	T	
rs9939609	*FTO*	0,388	0,436	0,407	0,426	0,421	0,41	A	

Supplementary Table 3.

Minor Allele Frequencies (MAF) in the case-control study populations according to the two GDM diagnostic criteria applied and in the European general population.
