# Supplementary material for: Association Study with 77 SNPs Confirms the Robust Role for the rs10830963/G of MTNR1B Variant and Identifies Two Novel Associations in Gestational Diabetes Mellitus Development
Source: PLoS One. 2017 Jan 10;12(1):e0169781. doi: 10.1371/journal.pone.0169781 (PMC5224877; doi:10.1371/journal.pone.0169781)
Supplement: S4 Table — (PDF) [file pone.0169781.s004.pdf]

SNP Id	Gene Name (HGNC Abbreviation)	Observed			Expected	
		BB (Homozygous for Major Allele)	Bb (Heterozygous)	bb (Homozygous for Minor Allele)	Expected BB	Chi-square test (0=non-deviant, 1=deviant)
rs10010131	*WFS1*	0.4214418	0.43438078	0.144177449	0.4078510	0
rs10423928	*GIPR*	0.5629860	0.36391913	0.073094868	0.5549439	0
rs1065780	*IGFBP1*	0.4305772	0.45865835	0.110764431	0.4354765	0
rs10738760	*VLDLR/KCNV2*	0.2832551	0.45383412	0.262910798	0.2602756	0
rs10811661	*CDKN2A/2B*	0.7045101	0.26127527	0.034214619	0.6974718	0
rs10830963	*MTNR1B*	0.4984520	0.39473684	0.106811146	0.4841661	0
rs10871777	*MC4R*	0.6021505	0.33486943	0.062980031	0.5922615	0
rs1111875	*HHEX/IDE*	0.4252336	0.42367601	0.151090343	0.4058603	0
rs11190604	*HIF1AN*	0.6194969	0.32704403	0.053459119	0.6131185	0
rs1143634	*IL1B*	0.5743034	0.37306502	0.052631579	0.5788713	0
rs11642841	*FTO*	0.3664596	0.43633540	0.197204969	0.3417891	0
rs1169288	*HNF1A*	0.3932584	0.44301766	0.163723917	0.3779388	0
rs11708067	*ADCY5*	0.6332574	0.32118451	0.045558087	0.6301973	0
rs11920090	*SLC2A2*	0.7834101	0.19815668	0.018433180	0.7787859	0
rs12243326	*TCF7L2*	0.4945736	0.42015504	0.085271318	0.4965333	0
rs12255372	*TCF7L2*	0.4738462	0.44000000	0.086153846	0.4814225	0
rs1227929	*SLC4A10*	0.4519685	0.37637795	0.171653543	0.4098016	0
rs12463617	*TMEM18*	0.6863354	0.28105590	0.032608696	0.6837030	0
rs12534093	*IGF2BP3*	0.6348228	0.31895223	0.046224961	0.6309108	0
rs1260326	*GCKR*	0.2830189	0.49056604	0.226415094	0.2791029	0
rs12779790	*CDC123, CAMK1D*	0.7305296	0.24299065	0.026479751	0.7259465	0
rs13266634	*SLC30A8*	0.5086614	0.41102362	0.080314961	0.5100434	0
rs1470579	*IGF2BP2*	0.5038880	0.38880249	0.107309487	0.4876079	0
rs1552224	*CENTD2*	0.7453125	0.24062500	0.014062500	0.7493066	0
rs17782313	*MC4R*	0.6064815	0.33487654	0.058641975	0.5989518	0
rs1799884	*GCK*	0.7087827	0.26040062	0.030816641	0.7038926	0
rs1800574	*HNF1A*	0.9449686	0.05345912	0.001572327	0.9441972	0
rs1801214	*WFS1*	0.4410339	0.41033926	0.148626817	0.4175790	0
rs1801282	*PPARG*	0.7647975	0.21183801	0.023364486	0.7581472	0
rs2286615	*BAD*	0.7711443	0.21393035	0.014925373	0.7710762	0
rs231362	*KCNQ1*	0.2955975	0.46540881	0.238993711	0.2791029	0
rs2464196	*HNF1A*	0.4746988	0.42168675	0.103614458	0.4699681	0
rs2867125	*TMEM18*	0.6913386	0.28503937	0.023622047	0.6953196	0
rs2871865	*IGF1R*	0.8059468	0.18779343	0.006259781	0.8097183	0
rs2890652	*LRP1B*	0.6810478	0.29429892	0.024653313	0.6859106	0
rs35767	*IGF1*	0.6692913	0.29448819	0.036220472	0.6667301	0
rs3741205	*IGF2*	0.5660848	0.24189526	0.192019950	0.4720135	0
rs41423247	*NR3C1*	0.4868624	0.40494590	0.108191654	0.4751833	0
rs4402960	*IGF2BP2*	0.4773791	0.42121685	0.101404056	0.4733268	0
rs4430796	*HNF1B(TCF2)*	0.3072197	0.46697389	0.225806452	0.2923636	0
rs4689388	*WFS1*	0.4066456	0.43670886	0.156645570	0.3906250	0
rs4712526	*CDKAL1*	0.4767081	0.40372671	0.119565217	0.4604592	0
rs4844880	*HSD11B1*	0.6881890	0.27401575	0.037795276	0.6809498	0
rs4846567	*SLC30A10*	0.5984127	0.34920635	0.052380952	0.5975535	0
rs4973768	*SLC4A7*	0.2741680	0.50871632	0.217115689	0.2793399	0
rs5015480	*HHEX/IDE*	0.4147105	0.43035994	0.154929577	0.3967620	0
rs5215	*KCNJ11*	0.4120444	0.42630745	0.161648177	0.3908727	0
rs5219	*KCNJ11*	0.4129032	0.42741935	0.159677419	0.3926437	0
rs571312	*MC4R*	0.6181534	0.32707355	0.054773083	0.6110395	0
rs5945326	*DUSP9*	0.5987362	0.35071090	0.050552923	0.5992178	0
rs6198	*NR3C1*	0.6674365	0.30023095	0.032332564	0.6683912	0
rs6832769	*CLOCK*	0.4467085	0.44984326	0.103448276	0.4510870	0
rs6884205	*TGFB2*	0.6358025	0.33179012	0.032407407	0.6427189	0
rs6905288	*VEGFA*	0.3322933	0.48673947	0.180967239	0.3313879	0
rs6921438	*VEGFA*	0.3012422	0.52018634	0.178571429	0.3150974	0
rs6993770	*ZFPM2*	0.5062500	0.41250000	0.081250000	0.5076563	0
rs720390	*IGF2BP2*	0.4122966	0.46292948	0.124773960	0.4144286	0
rs72865282	*AC092841.1 (miRNA)*	0.8183486	0.17064220	0.011009174	0.8166190	0
rs7310409	*HNF1A*	0.3266564	0.47765794	0.195685670	0.3197737	0
rs734312	*WFS1*	0.3555901	0.45186335	0.192546584	0.3381675	0
rs738409	*PNPLA3*	0.5978091	0.36619718	0.035993740	0.6098168	0
rs7501939	*TCF2*	0.4299065	0.42367601	0.146417445	0.4118361	0
rs757210	*HNF1B(TCF2)*	0.4345794	0.42990654	0.135514019	0.4218927	0
rs7578326	*lncRNA class RNA gene in the LOC646736/IRS1 region*	0.3981043	0.46287520	0.139020537	0.3963230	0
rs7608798	*DPP4*	0.4139194	0.47985348	0.106227106	0.4275148	0
rs7754840	*CDKAL1*	0.4750779	0.40809969	0.116822430	0.4612145	0
rs7756992	*CDKAL1*	0.5312500	0.37500000	0.093750000	0.5166016	0
rs780094	*GCKR*	0.2992126	0.48976378	0.211023622	0.2960388	0
rs7903146	*TCF7L2*	0.4713178	0.43100775	0.097674419	0.4717241	0
rs7950226	*ARNTL*	0.2848665	0.54302671	0.172106825	0.3095585	0
rs7957197	*HNF1A*	0.6785714	0.28726708	0.034161491	0.6760210	0
rs8191754	*IGF2R*	0.7769111	0.19968799	0.023400936	0.7686995	0
rs891088	*INSR*	0.5476923	0.38461538	0.067692308	0.5476000	0
rs900145	*ARNTL*	0.5269168	0.36541599	0.107667210	0.5035674	0
rs9341105	*IGFBP2*	0.5700787	0.36535433	0.064566929	0.5666415	0
rs9551419	*PDX1*	0.7058824	0.27399381	0.020123839	0.7104454	0
rs9939609	*FTO*	0.3596215	0.44006309	0.200315457	0.3359976	0

Supplementary Table 4.

Chi Squared test results for the assessment of the devation from the Hardy-Weinberg Equilbrium (HWE) genotype distributions.
