# Supplementary material for: Association Study with 77 SNPs Confirms the Robust Role for the rs10830963/G of MTNR1B Variant and Identifies Two Novel Associations in Gestational Diabetes Mellitus Development
Source: PLoS One. 2017 Jan 10;12(1):e0169781. doi: 10.1371/journal.pone.0169781 (PMC5224877; doi:10.1371/journal.pone.0169781)
Supplement: S5 Table — (PDF) [file pone.0169781.s005.pdf]

**Criteria**		**Austria**								n total (case/ctrl)
		Group and PG value (mmol/L) at 75g OGTT (24-28gw)				Group and BMI (kg/m^2)		Group and age (years)		333
		Case_0' min	Case_120' min	Ctrl_0'min	Ctrl_120'min	Case_BMI	Ctrl_BMI	Case_age	Ctrl_age	
IADPSG	mean	5,39	7,77*	4,41	5,67	27,77	24,11	31,7*	30,9	(103/230)
	SD	0,64	1,97	0,41	1,24	6,53	5,04	5,99	6,59	
m99'WHO	mean	5,2	8,9	4,58	5,54	27,35	24,65	32,37*	30,8	(73/260)
	SD	0,8	1,25	0,55	1,21	6,49	5,45	5,86	6,53	

		**Hungary**								n total (case/ctrl)
		Group and PG value (mmol/L) at 75g OGTT (24-28gw)				Group and BMI (kg/m^2)		Group and age (years)		584
		Case_0	Case_120	Ctrl_0	Ctrl_120	Case_BMI	Ctrl_BMI	Case_age	Ctrl_age	
IADPSG	mean	5,28	8,52*	4,45	5,83	26,66	23,64	33,9*	31,49	(114/440)
	SD	0,77	1,86	0,36	1,39	6,05	4,85	5,5	5,36	
m99'WHO	mean	5,02	8,92	4,52	5,53	26,4	23,59	33,8*	31,4	(166/418)
	SD	0,79	1,21	0,45	1,13	5,86	4,88	5,17	5,47	

Supplementary Table 5. The re-calculated mean clinical data of the Austrian and Hungarian pregnant populations.

The re-calculations were performed using both GDM diagnostic criteria (IADPSG and m'99WHO) in both countries.
* Significant differences between the clinical data of the Austrian and Hunagrian study populations.
The difference found in the 120'min plasma glucose values between the Austiran and Hungarian disease population may only be in part exlpained by their more advanced age (i.e. after adjustment to age the absolute value of Welch test t-value reflecting significance decreased from [-2.88] to [-2.5], but still remained significant (abs t-value>1.96)
